# Identification of the cuproptosis-related hub genes and therapeutic agents for sarcopenia

**DOI:** 10.3389/fgene.2023.1136763

**Published:** 2023-03-17

**Authors:** Yingqian Zhu, Xin Chen, Shasha Geng, Qingqing Li, Yang Li, Huixiao Yuan, Hua Jiang

**Affiliations:** ^1^ Department of Geriatrics, Shanghai East Hospital, Tongji University School of Medicine, Shanghai, China; ^2^ Department of General Medicine, Shanghai East Hospital, Tongji University School of Medicine, Shanghai, China

**Keywords:** sarcopenia, cuproptosis, mitochondrial metabolism, metformin, bioinformatics

## Abstract

**Background:** Along with acceleration of population aging, the increasing prevalence of sarcopenia has posed a heavy burden on families as well as society. In this context, it is of great significance to diagnose and intervene sarcopenia as early as possible. Recent evidence has indicated the role of cuproptosis in the development of sarcopenia. In this study, we aimed to seek the key cuproptosis-related genes that can be used for identification and intervention of sarcopenia.

**Methods:** The GSE111016 dataset was retrieved from GEO. The 31 cuproptosis-related genes (CRGs) were obtained from previous published studies. The differentially expressed genes (DEGs) and Weighed gene co-expression network analysis (WGCNA) were subsequently analyzed. The core hub genes were acquired by the intersection of DEGs, WGCNA and CRGs. Through logistic regression analysis, we established a diagnostic model of sarcopenia based on the selected biomarkers and was validated in muscle samples from GSE111006 and GSE167186. In addition, Kyoto Encyclopedia of Genes and Genomes (KEGG) and Gene Ontology (GO) enrichment analysis were performed on these genes. Furthermore, the gene set enrichment analysis (GSEA), and immune cell infiltration were also conducted on the identified core genes. Finally, we screened the potential drugs targeting the potential biomarkers of sarcopenia.

**Results:** A total of 902 DEGs and WGCNA containing 1,281 significant genes were preliminarily selected. Intersection of DEGs, WGCNA and CRGs yielded four core genes (PDHA1, DLAT, PDHB, and NDUFC1) as potential biomarkers for the prediction of sarcopenia. The predictive model was established and validated with high AUC values. KEGG pathway and Gene Ontology biological analysis indicated these core genes may play a crucial role in energy metabolism in mitochondria, oxidation process, and aging-related degenerative diseases. In addition, the immune cells may be involved in the development of sarcopenia through mitochondrial metabolism. Finally, metformin was identified as a promising strategy of sarcopenia treatment *via* targeting NDUFC1.

**Conclusion:** The four cuproptosis-related genes PDHA1, DLAT, PDHB and NDUFC1 may be the diagnostic biomarkers for sarcopenia, and metformin holds great potential to be developed as a therapy for sarcopenia. These outcomes provide new insights for better understanding of sarcopenia and innovative therapeutic approaches.

## Introduction

Sarcopenia is an age-related disease characterized by progressive loss of skeletal muscle mass and strength with decline in physical function (Cruz-Jentoft and Sayer, 2019). The aging individuals with sarcopenia are at higher risk of adverse outcomes, including frailty, impaired quality of life, falls, fracture, disability, and mortality (De Buyser et al., 2016; Schaap et al., 2018; Cruz-Jentoft and Sayer, 2019). The prevalence of sarcopenia increases with advancing age, which was approximately 10% for older people aged 60–70 years, and 30% for those over 80 years old (Cooper et al., 2012; Mijnarends et al., 2016). Sarcopenia is connected to skeletal muscle fibers loss and muscle atrophy. The muscle fibers are generally divided into slow-twitch type I muscle fiber and fast-twitch type II muscle fiber based on expression of different myosin isoforms and their contractile properties (Schiaffino and Reggiani, 2011). On the muscle fiber level, aging does not affect the muscle fiber types equivalently, and the loss of muscle fibers is not homogeneous (Tosetti et al., 2020). Skeletal muscle aging predominantly causes fast-twitch type II muscle fiber atrophy and fiber necrosis with decline in muscle strength, whereas type I fibers are less affected due to transition of muscle fibers from type II to type I with age (Hitachi et al., 2022). The reduction in the numbers of both two types of muscle fibers and specific type II muscle fiber atrophy, leading to the ageing-related phenotype known as sarcopenia. In addition, reduced number of type II satellite cells (Verdijk et al., 2014), together with progressive infiltration of fat and non-contractile tissue into muscle are also involved in the development of sarcopenia (Visser et al., 2005). Despite the rapidly increasing translation research in the therapeutic field of sarcopenia, exercise remains the primary treatment of sarcopenia (Dent et al., 2018), and no specific drug has been approved for the treatment of sarcopenia (Cho et al., 2022). Hence, it is of great significance to fully understand the potential mechanism and to develop new effective strategy for treating sarcopenia.

Copper is one of the essential trace elements in the human body playing several vital roles in living organisms. As an enzyme cofactor, copper is considerably involved in a broad range of biological processes, such as mitochondrial respiration, antioxidant defense, and iron metabolism (Ruiz et al., 2021). Copper also exerts its function as a dynamic signaling element with effects on the processes of lipolysis, cytotoxicity, cell proliferation, oxidative stress. In this regard, copper homeostasis is crucial for cell survival and metabolism. Copper is a redox active metal, the imbalance in its concentration and metabolism, is connected with the development of many diseases, including cancer, neurodegenerative diseases, metabolic syndrome, anemia and Wilson disease (Chen et al., 2020). Accumulating evidence suggests that copper toxicity disrupts mitochondrial metabolic enzymes, which induces a specific regulatory cell death mechanism defined as cuproptosis (Tang et al., 2022). Distinguishable from oxidative stress-related cell death, such as apoptosis, necroptosis and ferroptosis, cuproptosis is ignited by mitochondrial stress, especially the aggregation of mitochondrial enzymes lipoylation. The overload of copper can directly bind to lipoylated components of the tricarboxylic acid cycle in mitochondrial respiration, leading to the accumulating protein toxic stress and cell death (Tsvetkov et al., 2022). Current data has revealed the role of cuproptosis ignited by aging and genetic mutation in the progression of pathological sequelae, including neurodegenerative diseases, cancer, and inflammation (Gromadzka et al., 2020; Huang et al., 2022; Zhang et al., 2022; Zhao et al., 2022). The development of sarcopenia, characterized by age-associated with loss of skeletal muscle mass and strength, has been shown to be caused by dysregulated mitochondrial function, increased ROS generation and denervation (Johnson et al., 2013; Hood et al., 2019). However, at present, there are few studies on the cuproptosis and their association with sarcopenia. Therefore, in this study, we aim to explore the cuproptosis related biomarkers that can be used as prognostic indicators for sarcopenia, to further understand the potential mechanism of cuproptosis in the development of sarcopenia, which may improve the therapeutic strategies for prevention, diagnosis and treatment of sarcopenia in the future.

## Methods

### Data sources

The expression profiling generated by the high-throughput sequencing dataset GSE111016 was downloaded from Gene Expression Omnibus (GEO, https://www.ncbi.nlm.nih.gov/geo/). The GSE111016 expression profiling contained 40 muscle biopsy samples, including 20 subjects with sarcopenia and 20 healthy controls. The cuproptosis-related genes were extracted from previous published studies, which contained 31 cuproptosis-related genes (Supplementary Table S1) (Lin et al., 2022; Liu, 2022; Tsvetkov et al., 2022).

### Data processing

The raw count of GSE111016 is normalized by “edgeR”, and DEGs between sarcopenia and healthy control were identified *via* using the “limma” package [adjusted *p* value < 0.05 and log2 (fold change (FC)]≥ 0.3785. The DEGs with log FC < 0 were considered as down-regulated, while log FC > 0 were regarded as up-regulated. The volcano plots of DEGs and cluster analysis heat map were visualized *via* using “ggplot2” and “heatmap” packages in R software.

### Weighed gene co-expression network analysis

Weighed gene co-expression network analysis (WGCNA) is a bioinformatic method for the identification of the trait-related genes with importance (Langfelder and Horvath, 2008). The co-expression network for sarcopenia was constructed with “WGCNA” package in R. We removed the abnormal expressed genes from RNA-seq data of the training set by using goodSampleGenes method. The spearman’s correlation matrices and average linkage method were performed for all the pair-wise genes, and the similarity matrix was constructed by calculating the correlation coefficient between gene pairs. A suitable soft threshold power was set to transform the similarity matrix into an adjacency matrix to ensure the construction of a scale-free network. Subsequently, the adjacency was transformed into a topological overlap matrix (TOM) to measure the mean network connectivity of each gene, defined as the sum of its adjacency with all other genes for network generation, and then, the corresponding dissimilarity (1-TOM) was calculated to form clusters. According to the TOM-based dissimilarity measure with a minimum size of 30 for the genes dendrogram, average linkage hierarchical clustering was performed to classify co-expressed genes into different gene modules. To further analyze the module, we calculated the dissimilarity of module eigen (ME) genes, chose a cut line for module dendrogram and merged some module. We combined the modules with distance of less than 0.25, and finally obtained 14 co-expression modules shown in different colors. The module with the highest absolute value of the correlation coefficient was identified as the hub gene candidates for further analysis. The correlation coefficient representing the association between a gene module and sarcopenia phenotype, is considered significant with *p* value < 0.05.

### Hub genes identification and enrichment analysis

To further identify the sarcopenia related hub genes, we used the “VennDiagram” package to intersect the genes obtained by WGCNA and DEGs, as well as the cuproptosis-related genes. To explore the expression differences of the hub genes, the expression of each hub gene in two groups were validated and shown in box plots. To further understand the biological mechanism of the hub genes that are associated with sarcopenia, the Kyoto Encyclopedia of Genes and Genomes (KEGG) and Gene Ontology (GO) enrichment analysis were performed and visualized by “clusterProfiler” and “enrichplot” packages in R, the adjust *p* value < 0.05 was considered statistically significant.

### Verification of hub genes

In this study, according to the expression profile data of hub genes, a receiver operating characteristic curve (ROC) was established *via* “pROC” in R. The predictive sensitivity and specificity of the diagnostic model were evaluated by the area under the ROC curve (AUC). To further evaluate the diagnostic value of the hub genes in the training GSE111016 dataset, two independent datasets (GSE111006 and GSE167186) were retrieved from GEO to verify the robustness of the established model.

### Gene-set enrichment analysis

To investigate the biological role of the four hub genes in sarcopenia, the patients were categorized into high and low expression groups based on the median expression levels of these hub genes. The background gene set was obtained from MSigDB (Liberzon et al., 2015). The outcomes of the analysis were presented using the “enrichplot” package in the R software for visualization.

### Immune cell infiltration and correlation analysis

To explore the immune microenvironment in sarcopenia patients, the immune infiltrations of immune cells were analyzed using the CIBERSORT algorithm (Newman et al., 2015). A total of 17 immune cells was analyzed, including T cells, B cells, NK cells, monocytes, macrophages, neutrophils, and dendritic cells. Box plots were drawn to show the expression levels of 17 immune infiltrating cells. Spearman correlations were calculated to explore the association of different type of immune cells and were visualized *via* the ggplot2 package.

### Target drugs analysis

The Drug Gene Interaction database (DGIdb, https://dgidb.org/) was utilized to screen the potential drugs targeting cuproptosis-related genes in sarcopenia (Freshour et al., 2021). The 3D chemical structures of corresponding drugs were visualized and downloaded from the PubChem database (https://pubchem.ncbi.nlm.nih.gov/) (Wang et al., 2012). 3D structures of target proteins were retrieved from the RCSB Protein Data Bank (https://www.rcsb.org/) to explore the docking mode of drugs (Berman et al., 2000). The molecular docking with receptor protein and ligands acquired from the above steps was performed with ChimeraX 1.5 software (Pettersen et al., 2021). To further verify the binding properties of ligand to the target protein, AutoDock vina 1.1.2 and PyMOL 4.3.0 tools were used for perform docking simulation and visualization. The docking binding energy < 0 kcal/mol indicates that the receptor and ligand can bind spontaneously, and binding energy < −5.0 kcal/mol is considered to have an excellent binding activity.

## Results

### Identification of sarcopenia related RNAs

**FIGURE 1:**
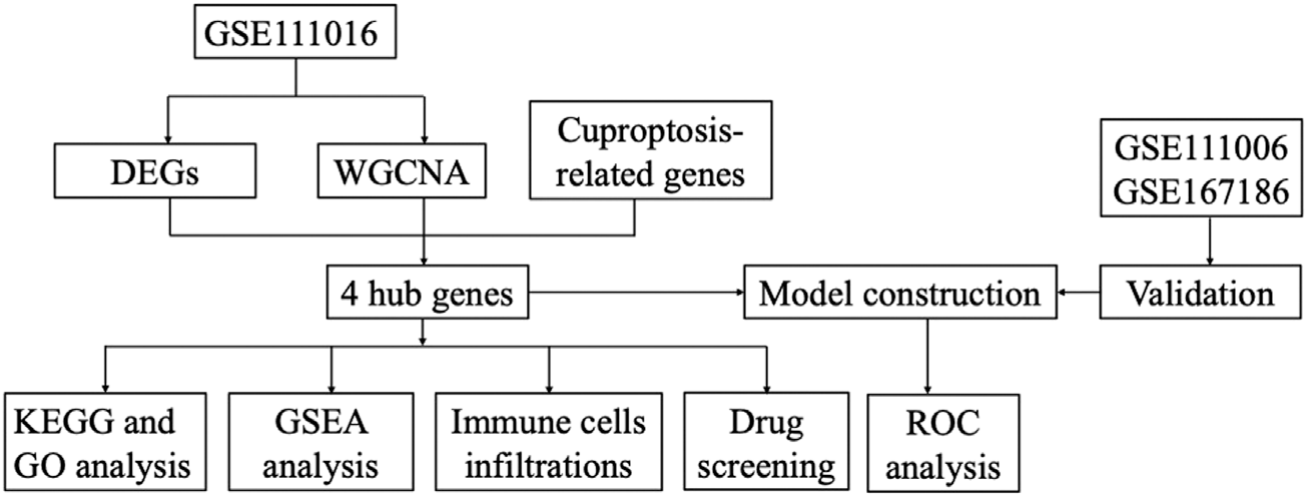
The flowchart of the study.

**FIGURE 2:**
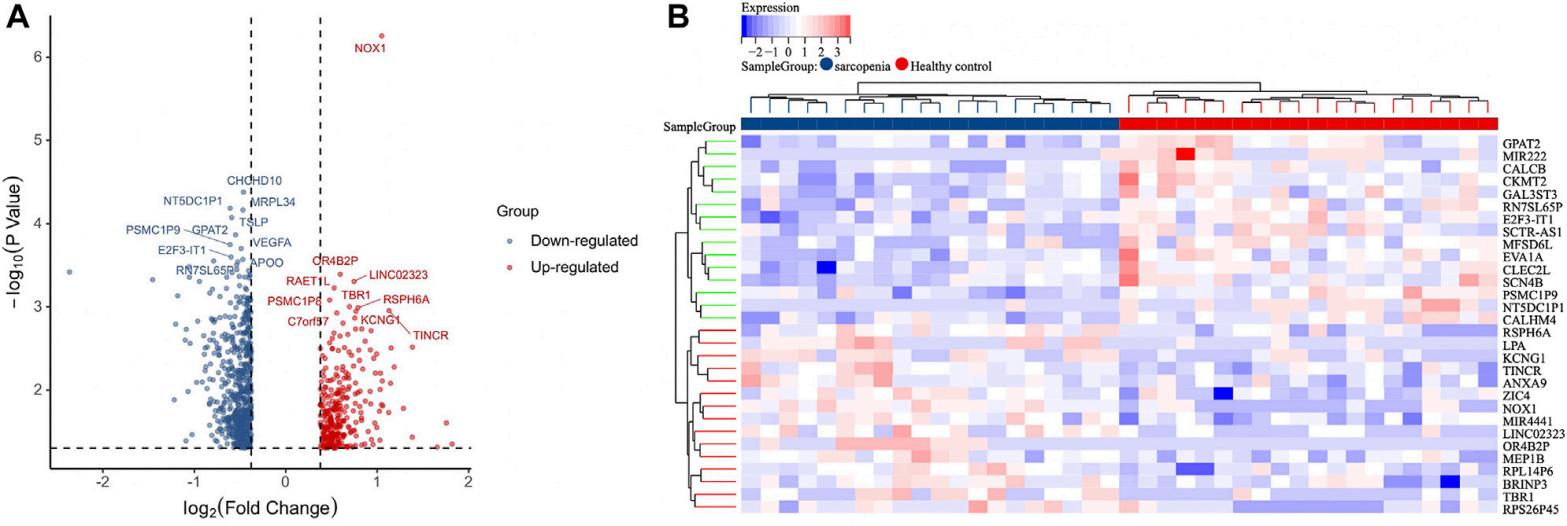
Identification of DEGs. **(A)** Volcano plot analysis of DEGs. Red color represents high expression, green color represents low expression. **(B)** The heatmap of top 30-fold-change DEGs. Red areas represent highly expressed genes and green areas represent lowly expressed genes involved in sarcopenia patients compared with healthy controls.

The gene expression profiles in vastus lateralis muscle of sarcopenia men and their age and race-matched corresponding healthy controls were retrieved from the GSE111016 dataset, which was further analyzed to investigate the biological functions of cuproptosis in the progression of sarcopenia. The age of healthy control was 69.95 ± 4.15 while the age of sarcopenia group was 72.75 ± 4.28, however, there was no statistical difference between the two groups. The detailed information of subjects in the GSE111016 was presented in Supplementary Table S2. A detailed flowchart of the study was showed in Figure 1. A total of 31,623 RNA-seq were obtained from GSE111016 database, and 902 DEGs were preliminary screened according to *p* value < 0.05 and FC ≥ 1.3, of which 288 were upregulated and 614 were downregulated. The DEGs are shown in the volcano plot (Figure 2A), the top 30 DEGs were plotted with a heatmap (Figure 2B).

### Construction and analysis of gene co-expression network

**FIGURE 3:**
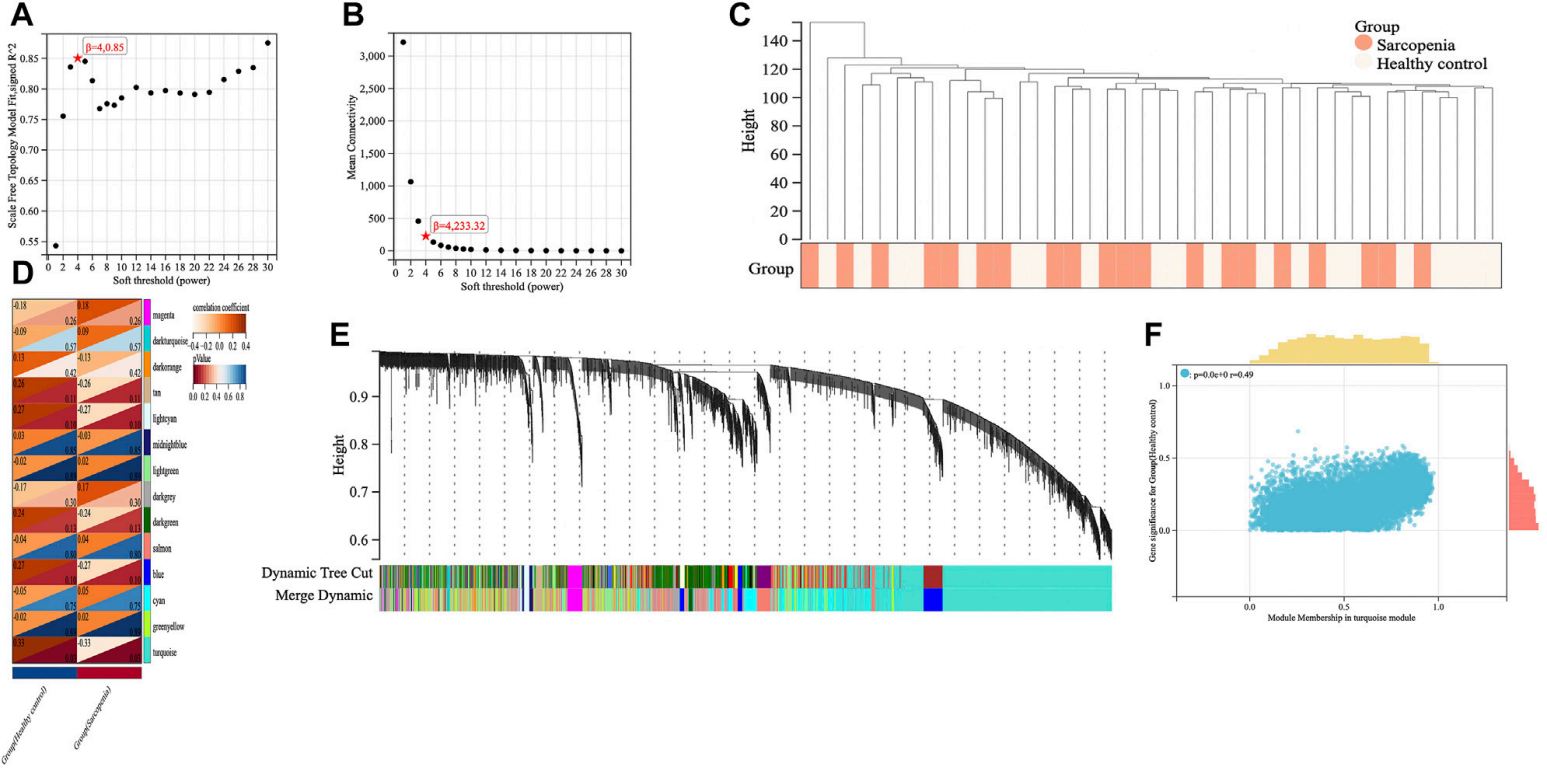
Screening of key genes by weighted gene co-expression network analysis (WGCNA). The scale independence **(A)** and the mean connectivity **(B)** to identify the soft threshold with best performance. **(C)** Clustering dendrogram and trait heatmap of samples. **(D)** The heatmap displaying the correlation between gene modules and clinical traits. The rows represent gene modules and columns correspond to clinical traits. Each cell contains the correlation coefficient (upper number) and *p* value (lower number). **(E)** Hierarchical cluster dendrogram and color-coding of gene co-expression modules. **(F)** Scatter plot showing the relationship between gene significance and module membership in the turquoise module.

We applied WGCNA analysis on the data of GSE111016 dataset to build the co-expression network. In WGCNA analysis, the soft thresholding power (β = 4) was select to ensure a relatively balanced scale independence and mean connectivity (Figures 3A,B). No outlier samples were excluded by sample cluster analysis (Figure 3C). In total, 14 gene modules distinct were generated through hierarchical clustering tree, in which each tree branch represented a module, and each leaf constitutes a gene in the dendrogram (Figure 3E). The correlations between modules and sarcopenia were computed. Among the modules, the turquoise module was selected as the most significant module correlated with sarcopenia (r = 0.33 and *p* = 0.03) (Figure 3D). Furthermore, the 1,281 genes were obtained in the turquoise module, which had the most remarkable correlation with both module and sarcopenia phenotype, the correlation was 0.49 and *p* value was less than 0.001, which indicated a strong relationship between these genes and clinical traits (Figure 3F).

### Identification and functional enrichment analysis of hub genes

**FIGURE 4:**
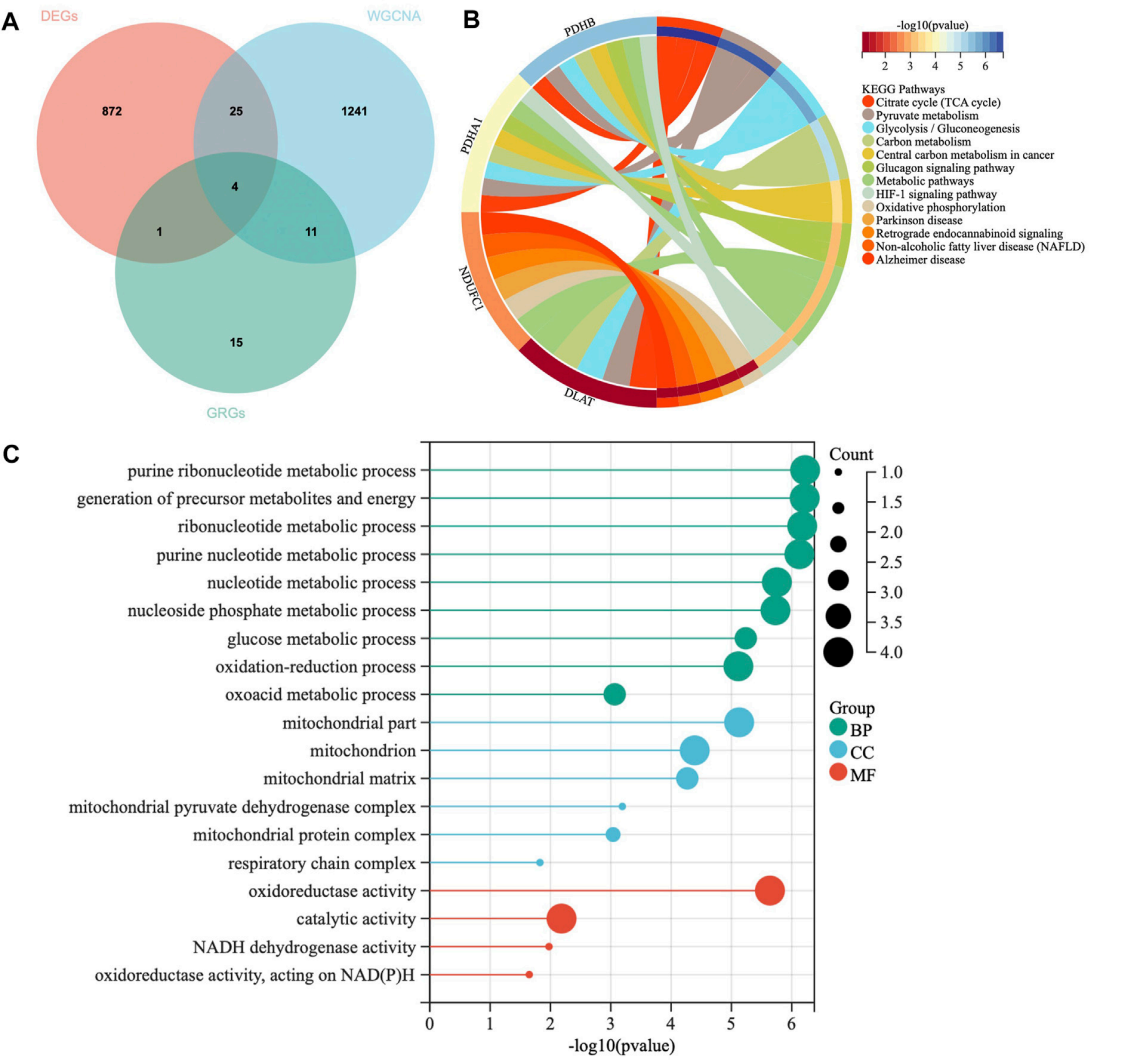
Identification of hub genes and Functional enrichment analysis. **(A)** The Venn diagram of genes among differentially expressed genes (DEGs) list, WGCNA and cuproptosis related genes (CRGs). **(B)** Kyoto Encyclopedia of Genes and Genomes (KEGG) enrichment analysis for hub genes. **(C)** Gene Ontology (GO) enrichment analysis for hub genes, including biological processes (BP), cellular components (CC) and molecular functions (MF).

A total of 31 cuproptosis-related genes (CRGs) were summarized from previous published studies. To identify the hub genes associated with sarcopenia and cuproptosis, a venn diagram was utilized to screen the genes between the DEGs, WGCNA and CRGs. As shown in Figure 4A, a total of four overlapping genes were extracted as the hub genes involved in sarcopenia and cuproptosis, which were PDHA1, DLAT, PDHB and NDUFC1. Further, the functional enrichment analysis was conducted to explore the functions and pathways of the four hub genes *via* clusterProfiler R package. Pathway enriched by KEGG were mainly related to RNAs with citrate cycle, glucagon and metabolic pathway, HIF-signaling pathway, retrograde endocannabinoid signaling, and several ageing related degenerative diseases (Figure 4B). GO enrichment analysis revealed that the four overlapping genes were enriched in multiple biological processes (BP) of metabolism, including ribonucleotide metabolic process, glucose metabolic process, generation of precursor metabolites and energy, oxidation reduction process and oxoacid metabolic process. With regards to cellular components (CC), the hub genes were associated with mitochondrion, mitochondrial matrix, mitochondrial pyruvate dehydrogenase complex and respiratory chain activity. Concerning molecular functions (MF), they were involved in oxidoreductase activity, catalytic activity and NADH dehydrogenase activity (Figure 4C).

### Construction and verification of multigene diagnostic model

**FIGURE 5:**
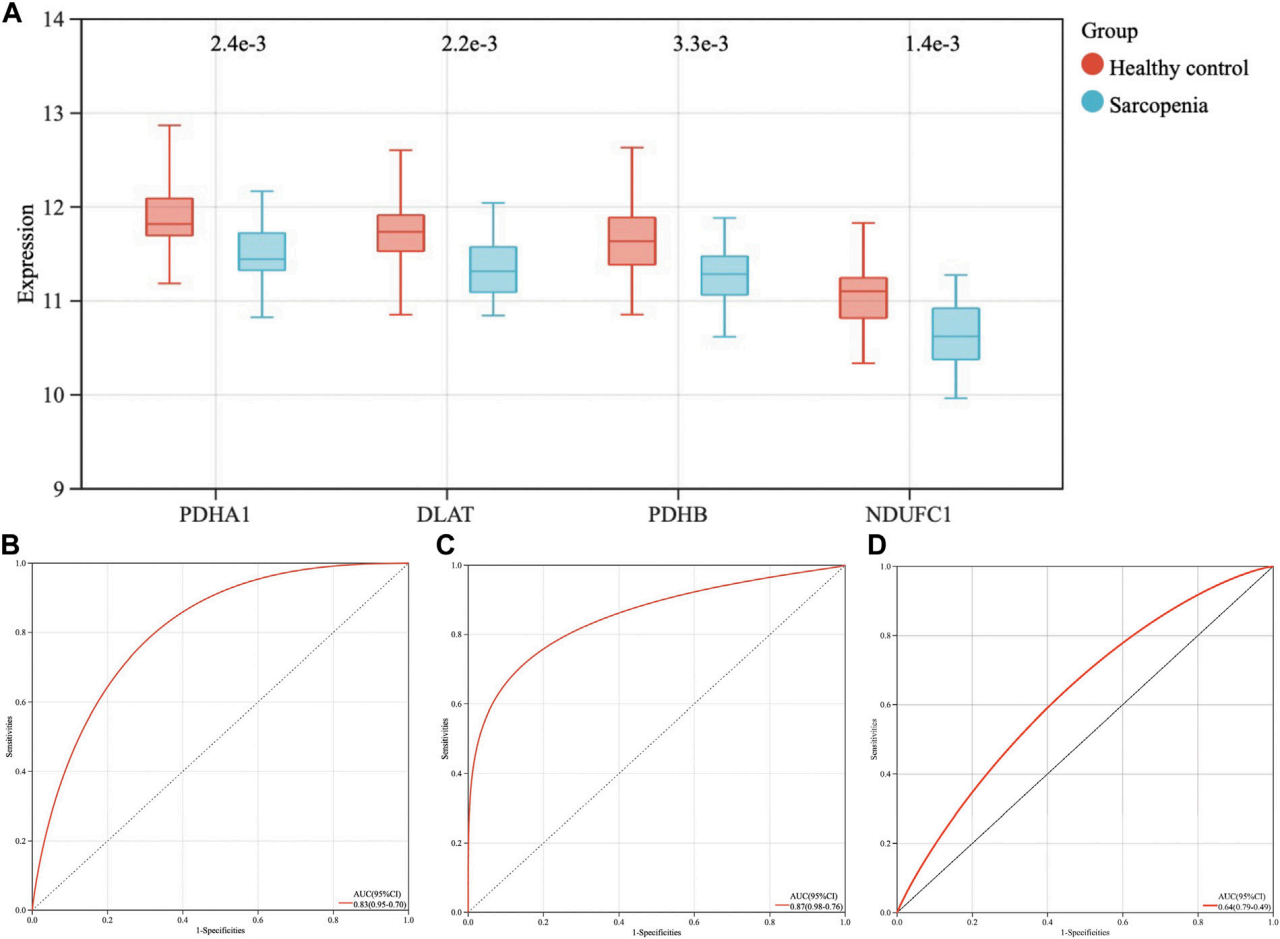
The expression levels of four hub genes, the establishment and validation of the diagnostic model for sarcopenia. **(A)** The expression levels of PDHA1, DLAT, PDHB, and NDUFC1 in sarcopenia group were downregulated in GSE111016 dataset. **(B)** Receiver operating characteristic (ROC) curve and area under the curve (AUC) statistics of predictive model of sarcopenia in GSE111016 dataset. ROC curves and AUC statistics to assess diagnostic efficiency of hub genes on the identification of sarcopenia in GSE111006 **(C)** and GSE167186 dataset **(D)**.

The expression levels of the four hub genes were demonstrated *via* box plots. As is showed in Figure 5A, the expression levels of PDHA1 (*p* = 2.4e-3), DLAT (*p* = 2.2e-3), PDHB (*p* = 3.3e-3) and NDUFC1 (*p* = 1.4e-3) in the sarcopenia group were significantly decreased than these in the healthy controls. Then, the ROC curve analysis of multigene prediction model of sarcopenia was constructed *via* logistic regression. To evaluate the sensitivity and specificity for the prediction model, the area under the curve (AUC) values of the four hub genes were calculated with the value of 0.83, which indicated the high predictive value of the hub genes for sarcopenia (Figure 5B). To further confirm the utility of this model, the diagnostic value of the four hub genes was validated in two independent datasets subsequently, with AUC values of 0.87 in GSE111006 dataset and 0.64 in GSE167186 dataset (Figures 5C,D). The sensitivity and specificity indicated the four genes had good diagnostic efficiency in sarcopenia.

### GSEA analysis of hub genes

**FIGURE 6:**
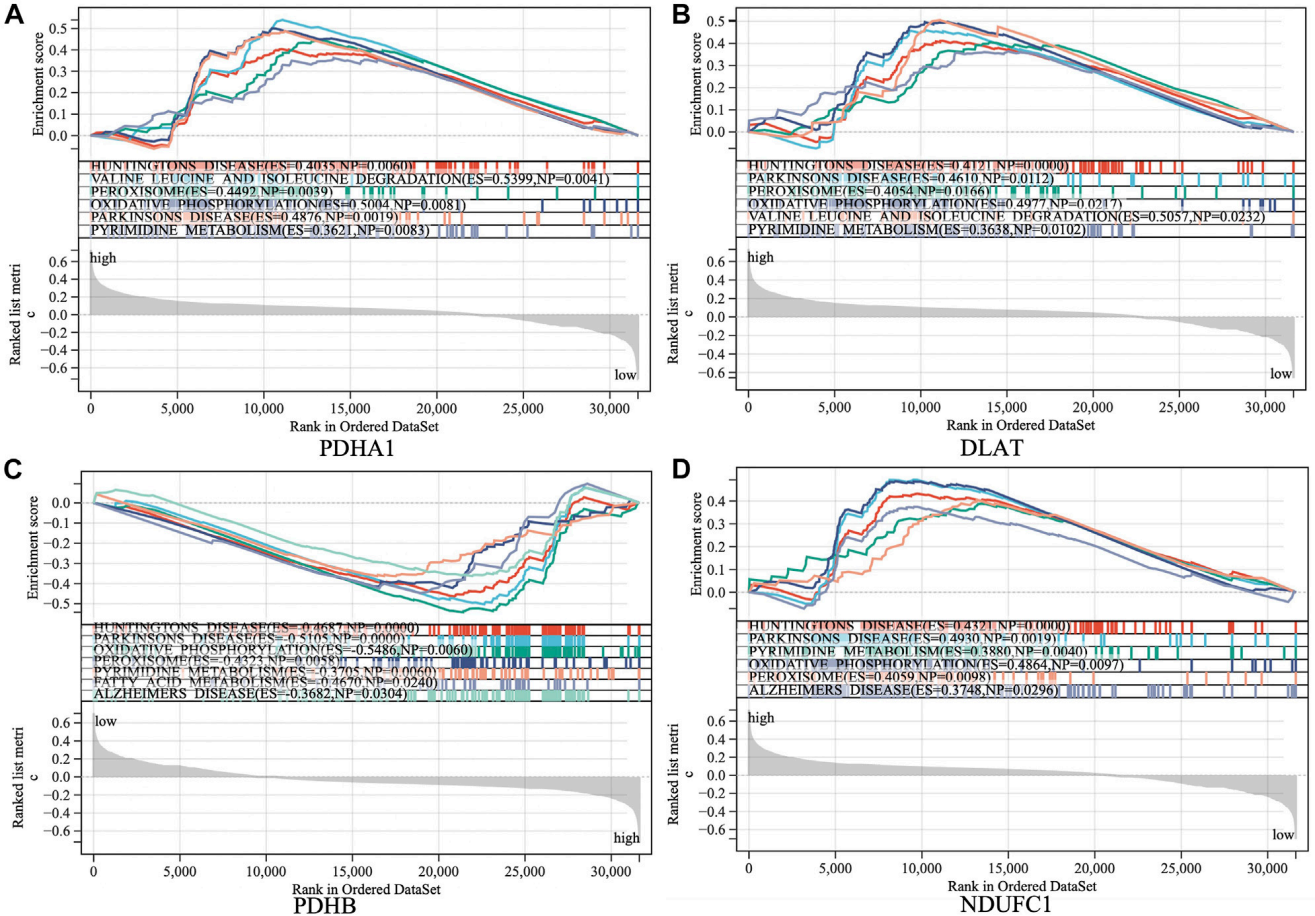
Gene set enrichment analysis (GSEA) to identify the enriched pathways of the central hub genes. **(A)** PDHA1; **(B)** DLAT; **(C)** PDHB; **(D)** NDUFC1.

The samples were divided into high/low expression groups of PHDA1, DLAT, PDHB and NDUFC1 for GSEA analysis, respectively. GSEA function and pathway analysis indicated that the four hub genes were significantly associated with several neurodegenerative diseases, which included Huntington diseases, Parkinson diseases, Alzheimer diseases. In addition, pathways related to several biological metabolic processes, such as oxidative phosphorylation, peroxisome, and pyrimidine metabolism, were enriched in the high expression levels of hub genes (Figure 6).

### Immune cell infiltration and correlation analysis

**FIGURE 7:**
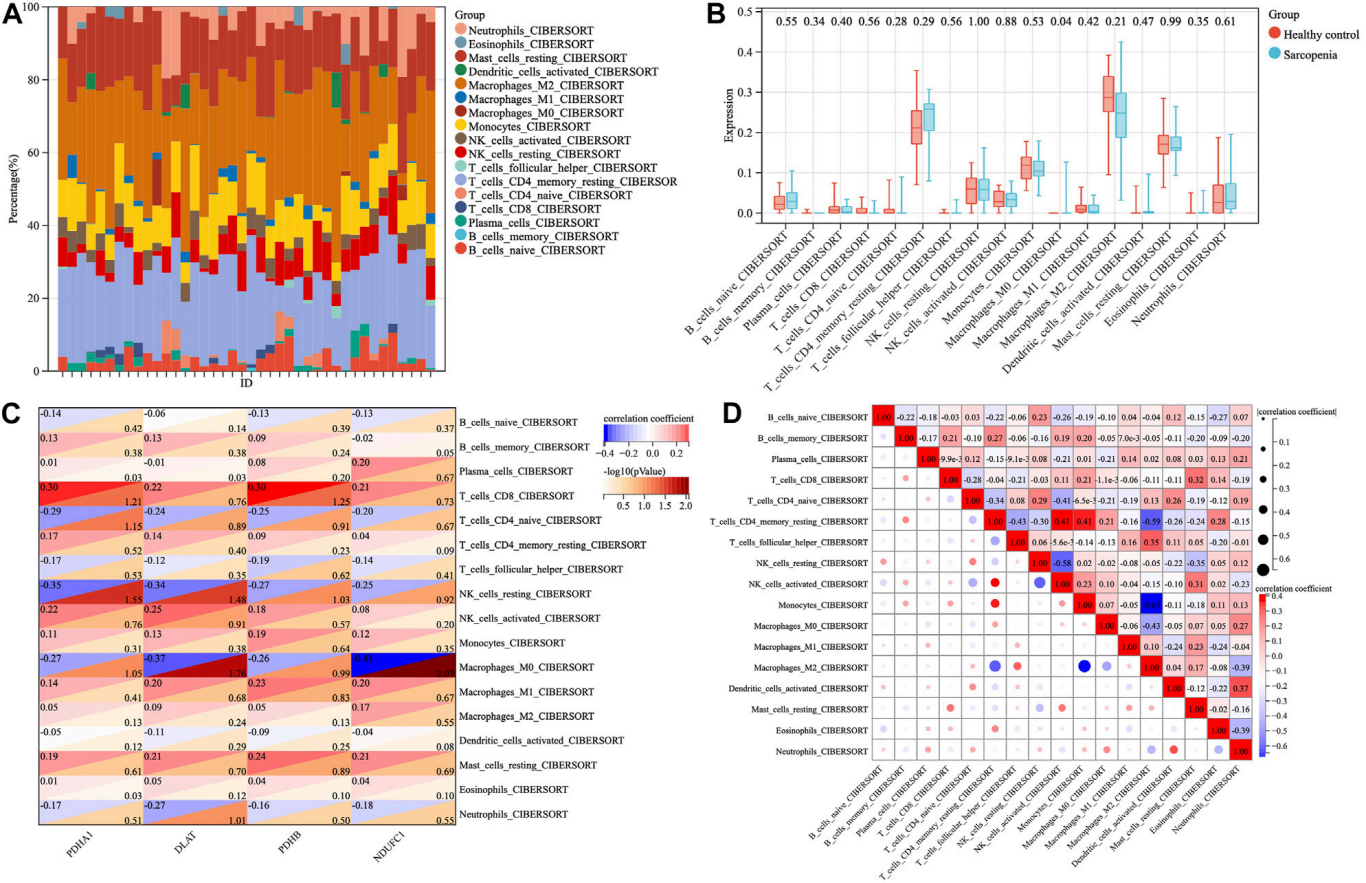
The proportions and correlations of immune cells among sarcopenia group and healthy control group. **(A)** The relative abundance of 17 distinct immune cells subsets. **(B)** The expression levels of immune cells shown in box plots among sarcopenia group and healthy control group, *p* < 0.05 indicated statistical difference. **(C)** The spearman’ correlation of each hub gene with different immune cell subsets. The rows correspond to immune cell subsets and each columns represents a hub gene. Each cell contains the correlation coefficient (upper number) and -log10 (*p* value) (lower number). **(D)** The correlations between different populations of immune cell subsets.

To investigate the immune microenvironment of sarcopenia, the relative abundance of several immune cell subtypes was calculated according to the transcriptome of all samples. The result of CIBERORT indicated that M2 macrophages, CD4^+^ memory T cells, and resting mast cells were the main infiltrative immune cells (Figure 7A). The infiltration of immune cells was compared between sarcopenia and healthy control groups (Figure 7B). In comparison with healthy control group, the sarcopenia group had higher fraction of M0 macrophages (*p* = 0.04), however, the proportions of other immune cell subsets showed no significant difference between the two groups. Subsequently, we evaluated the correlation between the expression of hub genes and infiltration levels of different cell subsets (Figure 7C). The results illustrated that PDHA1, DLAT, PDHB and NDUFC1 were negatively correlated with M0 macrophages and resting NK cells, were positively correlated with CD8+ T cells. Among them, NDUFC1 had the strongest association with M0 macrophages (r = −0.41, *p* = 0.0085). Next, the correlation between the immune cell populations was analyzed (Figure 7D). There was a strong negative correlation between M2 macrophages and monocytes (r = −0.65). Besides, the negative association between M2 macrophages and resting CD4+T cells was obviously observed (r = −0.59). The resting CD4+T cells were positively correlated with both activated NK cells and monocytes (r = 0.41).

### Prediction of potential therapeutic drugs

**FIGURE 8:**
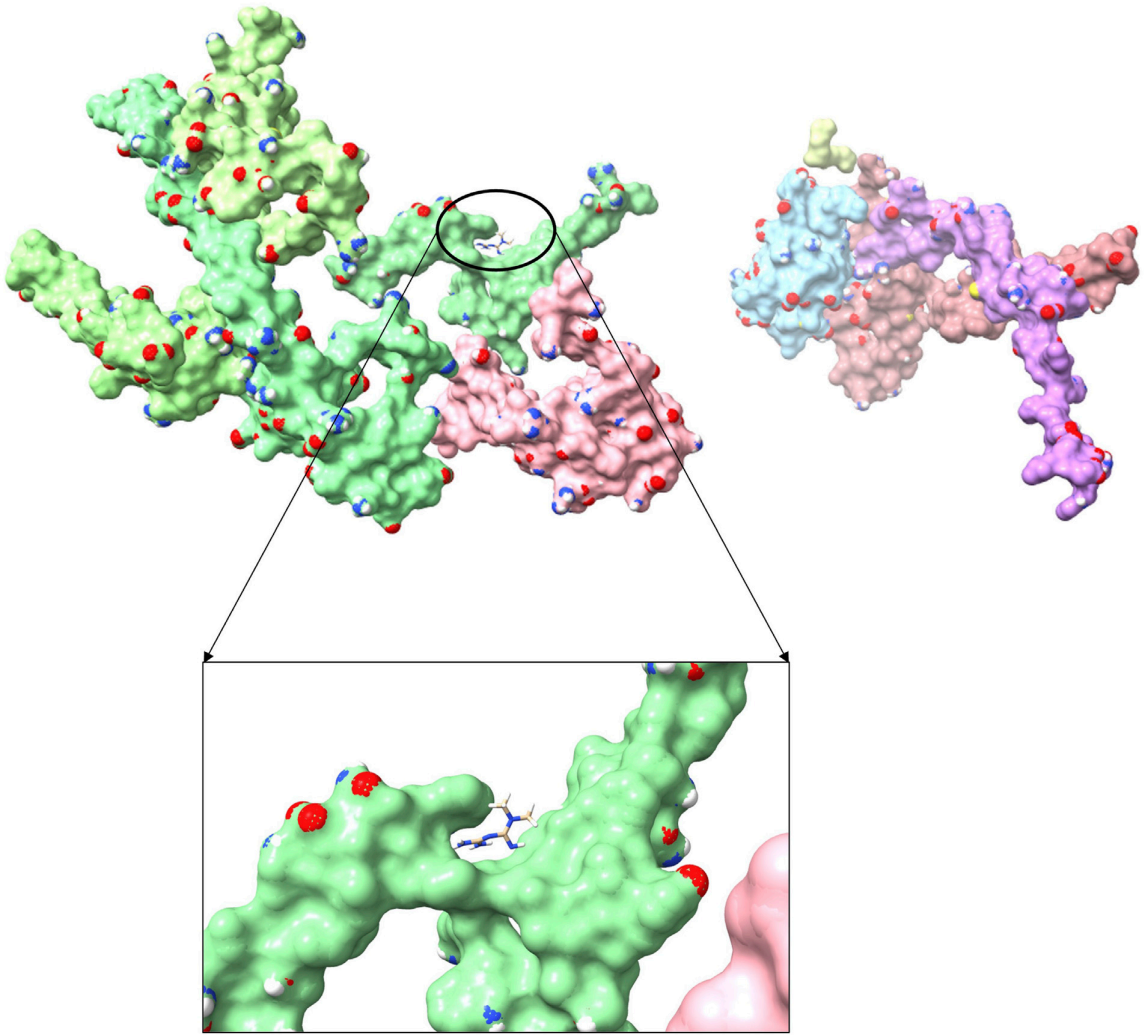
The three-dimensional molecular docking diagram of metformin and target protein NDUFC1.

**FIGURE 9:**
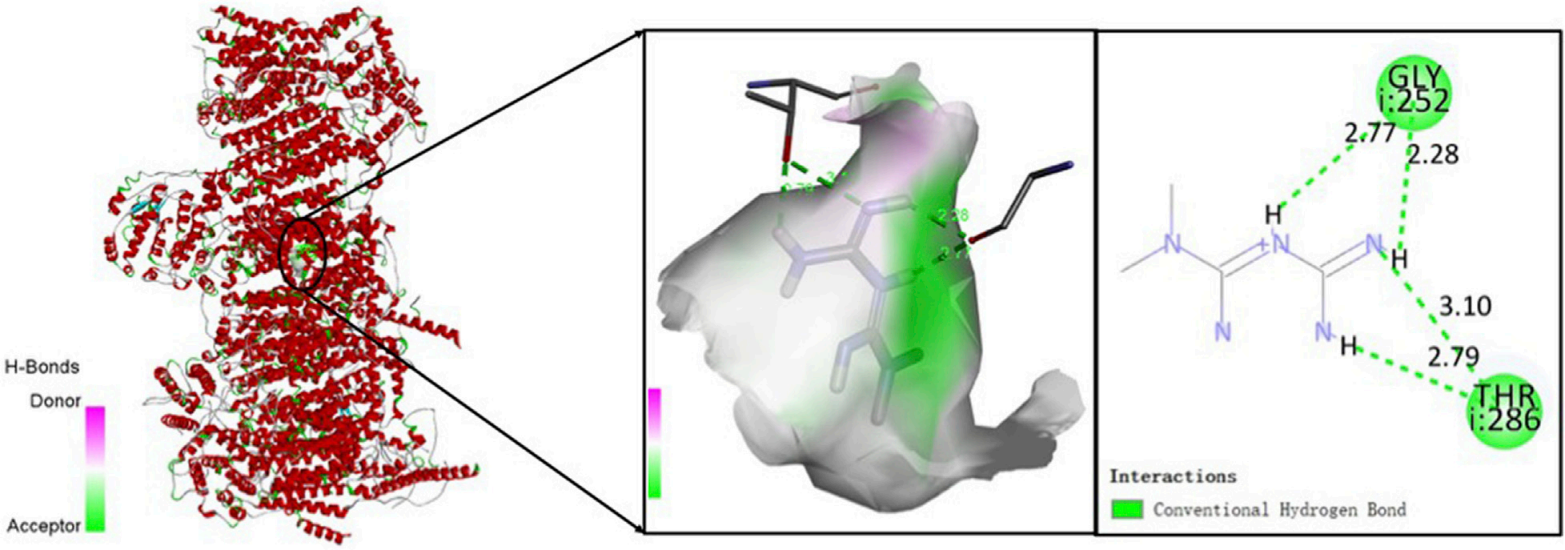
The two-dimensional molecular docking diagram of metformin and target protein NDUFC1. Hydrogen bonds are shown as green lines, and hydrophobic forces are denoted as pink color.

To explore the potential anti-sarcopenia medicines, drugs that may target on the cuproptosis-related hub genes were screened based on the DGIdb database. According to the findings, metformin hydrochloride was found to be the therapeutic medicine of NDUFC1, while no potential drugs was found for the other three genes (PDHA1, DLAT and PDHB). The 3D chemical structure of metformin hydrochloride was obtained from PubChem database for drug docking analysis. Simultaneously, the structure of target protein NDUFC1were retrieved from PCSB PDB database (PDB ID: 5XTC). Then, the molecular docking with receptor and ligand was carried out through ChimeraX 1.5 software (Figure 8). The docking binding energy was −43.968 kcal/mol, which displayed an excellent binding affinity of drug to the target protein. In order to investigate the binding mechanism of the small molecule metformin to the NDUFC1 protein, Autodock and PyMOL tools were used to perform molecular docking simulations. The binding energy between protein NDUFC1 and metformin is −5.3 kcal/mol, which revealed metformin exhibit a strong affinity for NDUFC1. In the two-dimensional plot, hydrogen bonds are depicted as green dashed lines (Figure 9). The results showed that metformin formed two hydrogen bonds with the 106th SER amino acid residue of the 5j3j protein, with bond lengths of 3.10 Å and 2.80 Å, as well as two hydrogen bonds with the 286th THR amino acid residue of the same protein, with bond lengths of 2.79 Å and 3.10 Å. These interactions, which were revealed through the analysis of the crystal structure, were critical for the stable binding of metformin to NDUFC1. These findings provide novel insight into the molecular interactions that enable metformin to modulate the activity of target protein.

## Discussion

Copper is an essential catalytic cofactor in the human body, which plays a crucial role in several biological processes ranging from oxygen metabolism to cell growth (Chen et al., 2022). In general, intracellular copper is maintained at relatively low concentration, the overload of copper can cause cytotoxicity and ultimately lead to cuproptosis, which is a recently identified form of regulated cell death (RCD) (Tang et al., 2022; Tsvetkov et al., 2022; Wang et al., 2022b). Distinct from the traditional oxidative stress-related cell death such as apoptosis, necroptosis and ferroptosis, cuproptosis is triggered by mitochondrial proteotoxic stress when excessive copper tightly binds to lipoylated proteins of tricarboxylic acid (TCA) cycle, followed by the aggregation of lipoylated mitochondrial enzymes in TCA and loss of Fe-S cluster proteins (Cobine and Brady, 2022; Li et al., 2022; Tsvetkov et al., 2022). The current findings have indicated that the copper imbalance contributed to skeletal muscle mitochondrial defects, which accelerate the development of muscle atrophy and weakness, and is a major initiator of sarcopenia (Su et al., 2021). However, the connections between cuproptosis and sarcopenia remains unknown. Therefore, we aimed to elucidate the specific mechanism of cuproptosis in this aging muscle phenotypes. In addition, gene signatures were used to identify the predictive model and potential therapeutic strategy with drugs to combat sarcopenia.

In this study, we comprehensively analyzed the expression profiles of muscle tissue between sarcopenia patients and healthy individuals. Totally, 902 DEGs were identified to be associated with the development of sarcopenia. WGCNA was employed to explore the highly correlated mRNA with clinical traits. To further obtain the cuproptosis-related hub genes, we took intersections of DEGs, key WGCNA modules, and cuproptosis related genes. A total of four possible hub genes of sarcopenia were screened, namely, PDHA1, DLAT, PDHB, and NDUFC1. The GO enrichment analysis revealed that these hub genes were predominantly involved in the energy and nucleotide metabolism related biological processes. Similarly, GO cellular component analysis showed the high enrichment of these genes for mitochondrial structures and mitochondrial respiration. The GO molecular function terms also highlighted their important roles in oxidoreductase activity, catalytic activity as well as NADH dehydrogenase activity, which is consistent with the perspective of earlier studies that dysfunctional mitochondria play a key role in initiating and exacerbating sarcopenia (Alway et al., 2017). It is well acknowledged that mitochondrial dysfunction is one the hallmarks of aging with the presence of decline in mitochondrial respiration and increase in oxidative stress (López-Otín et al., 2023). In our study, the GSEA analysis confirmed that the four hub genes were also involved in the age-related neurodegenerative diseases and cellular mitochondrial functions in skeletal muscles. This finding is in line with the previous evidence that the aging myoblasts isolated from old individuals showed reduced mitochondrial respiration with increased glycolysis, and the alternations in energy metabolism in muscle tissues during aging may be the leading causes of unhealthy aging and aging-related diseases (Sebastián et al., 2016).

Previous studies have suggested that these genes were functionally enriched in processed related to metabolism and cell activities such as cellular respiration and oxidation-reduction process (Yang et al., 2021). KEGG analysis showed that they were involved in biological processes related to mitochondrial functions in skeletal muscles, including central carbon metabolism, citrate cycle, glycolysis, and oxidative phosphorylation (Chae et al., 2018). In this study, we also discovered that the four hub genes were enriched in the age-related diseases, such as Alzheimer’s disease and Parkinson’s diseases. Previous researches have suggested that cuproptosis plays a pathogenic role in the progression of neurodegenerative diseases in the context of aging (Bandmann et al., 2015; Lai et al., 2022). Cognitive impairment and declining physical performance resulting from neurodegenerative diseases have been linked to an increased prevalence and severity of sarcopenia (Tay et al., 2018; Yazar et al., 2018; Yun et al., 2021). It is widely recognized that mitochondria play a crucial role in regulating energy metabolism, cell differentiation and death. Mitochondria function in muscle cells and motor neurons deteriorate with aging (Calvani et al., 2013; Rygiel et al., 2014), and crosstalk between these two compartments may promote the death of both (Alway et al., 2017).

The four hub genes (PDHA1, DLAT, PDHB, and NDUFC1) were included to construct a diagnostic model for sarcopenia. GSE111006 dataset was subsequently utilized to validate the predictive model. The AUC values in the ROC analysis were 0.83 in the training cohort and 0.87 in the external validation cohort, demonstrating the reliable and efficient prediction performance of model. According to the results of ROC analysis, PDHA1, DLAT, PDHB, and NDUFC1 were selected as the potential promising diagnostic biomarkers of sarcopenia. These four hub genes, are involved in metabolic pathways known as cellular respiration, playing crucial roles in cellular energy metabolism. PDHA1 and PDHB are major subunits of the pyruvate dehydrogenase complex, which can convert pyruvate, the product of glycolysis, into acetyl-CoA during oxidative phosphorylation. Pyruvate can enter the citric acid cycle to generate ATP, the primary energy source for the cells (Gottlieb and Bernstein, 2016). PDHA1 and PDHB were downregulated in sarcopenia patients, which indicated the dysfunction of mitochondrial respiration in sarcopenia patients. Accumulating evidence has implicated that mitochondrial dysfunction may underlie a growing number of neurodegenerative diseases and even the process of ageing (Tatsuta and Langer, 2008; Burté et al., 2015; Islam, 2017). In this study, we also found that expression levels of DLAT was downregulated in the sarcopenia group. DLAT is an enzyme component of pyruvate dehydrogenase complex in mitochondria that acts as an acetyltransferase catalyzing the transfer of an acetyl group from acetyl-CoA to lipoamide, which ensures the energy generated from pyruvate to be properly utilized (Alvarez-Calderon et al., 2015). The mutations in DLAT genes lead to energy deficiency in the cell, has been demonstrated to propagate various human diseases, such as movement and metabolic disorders (Méneret et al., 2021). NDUFC1 is an essential component of NADH dehydrogenase (Complex I) in the electron transport chain required for the structural and functional stability of the complex. NDUFC1, a key gene related to cuproptosis, is also downregulated among the sarcopenia subjects. NDUFC1 plays a crucial role in the transfer of electrons from NADH to ubiquinone, contributing to maintain the energy balance in the cells. According to the previous evidence, the knockdown of NDUFC1 can inhibit complex I function and enhance ROS generation (Han et al., 2022), which may promote the accumulation of mtDNA mutations. The mtDNA damage to sarcopenia and corresponding impaired function may be implicated in the significantly reduced complex I activity in aging skeletal muscles (Marzetti et al., 2009; Pestronk et al., 2017). The present study inferred the significance of mitochondrial dysfunction in the progression of sarcopenia, and the four cuproptosis-related genes, PDHA1, DLAT, PDHB, and NDUFC1, may serve as the potential targets for further therapeutic intervention. Currently, the precise mechanism of the aberrant expression of the four hub genes in promoting sarcopenia progression remains to be addressed.

The intramuscular immune cells may be involved in the progression of sarcopenia (Villalta et al., 2009; Wang et al., 2022a). In this study, we further analyzed the immune cell infiltration and the correlation of hub genes with immune factors. For most immune cell infiltration, there were no significant differences except for the quantity of M0 macrophages. The correlation between PDHA1, DLAT, PDHB, NDUFC1 and immune cells suggested that these hub genes were associated with CD8^+^ T cells, M0 macrophages, and resting NK cells. Evidence has indicated that the immune senescence plays a role in the onset of sarcopenia (Nelke et al., 2019). The immune system undergoes senescence with drastic changes, characterized by thymic involution, the accumulation of senescent T cells, and impaired function of innate immune cells, such as macrophages and NK cells. Age-dependent thymic atrophy significantly reduces the output of thymocytes from thymus, ultimately leading to impairment of T cell-mediated immunity and an increase in the proportion of CD8^+^ T cells, a major cause of sarcopenia (Oh et al., 2021). Macrophages that infiltrate skeletal muscle are responsible for the regeneration and inflammatory responses of skeletal muscle tissues. M0 macrophages are non-differentiated immune cells that can polarize into both pro-inflammatory M1 macrophages and anti-inflammatory M2 macrophages (Murray, 2017). In aged muscle, macrophages immunophenotyping reveals divergent muscle polarization. The dysregulated macrophage response during recovery from disuse corresponded to impaired muscle growth in old adults (Reidy et al., 2019). Consequently, the impaired activity of immunity in old age may contribute to both skeletal muscle atrophy and inflammatory responses. Additional research is required to fully comprehend the intricate interplay between immune cells and skeletal muscle cells.

Molecular docking, currently performed in structure-based virtual screening campaigns *via* rationalizing ligands activity towards a targeted protein, is a powerful tool in drug discovery (Pinzi and Rastelli, 2019). In this study, the molecular docking results suggested that metformin could be a potential therapeutic drug for sarcopenia by targeting NDUFC1. Metformin, a well-established anti-diabetes drug for decades, has recently shown the anti-aging effects extend longevity in different organism (Kulkarni et al., 2020). Metformin has been identified as an inhibitor of mitochondrial oxidative phosphorylation through inhibiting complex I, which can reduce the production of ROS and DNA damage in senescent cells (Ngoi et al., 2021). Metformin partially inhibiting mitochondrial respiratory complex I activity has been demonstrated to prevent mitochondrial dysfunction (Geng et al., 2020). A growing body of epidemiological evidence has indicated that T2DM patients receiving metformin had less decline in walking speed than those not taking (Lee et al., 2013). Similarly, the T2DM patients using metformin lost less muscle mass than controls (Lee et al., 2011), suggesting metformin may play a beneficial role in sarcopenia (Massimino et al., 2021). In this study, the drug analysis showed that metformin is a potential therapeutic agent on sarcopenia *via* binding to NDUFC1, which may have significant implications for possible therapeutic interventions on sarcopenia, however, the precise mechanism by which metformin act remains further research.

As with all studies, this study has several limitations. First, the transcriptome datasets of sarcopenia were available in only a limited number. In the present study, we performed the analyses with one training dataset and one validation set, which is the main limitation of the study. Second, despite we performed detailed analysis with application of bioinformatics technology, such as differential gene expression analysis, WGCNA, and biological pathway enrichment analysis, the key genes that may drive the progression of sarcopenia still be missed. Third, the causal mechanism of cuproptosis-related hub genes and immune cell infiltration that underlie these associations and lead to sarcopenia, still needed a large number of experiments to demonstrate.

## Conclusion

Our findings indicate the cuproptosis-related genes, PDHA1, DLAT, PDHB, and NDUFC1 are the diagnostic biomarker for sarcopenia, which provide evidence concerning the role of cuproptosis in sarcopenia. We also discover that metformin, the novel anti-aging drug, may be a potential therapeutic agent for the treatment of sarcopenia.

## Data Availability

The original contributions presented in the study are included in the article/Supplementary Material, further inquiries can be directed to the corresponding author.
